# 

**Published:** 1879-02-20

**Authors:** 


### TABLE OF CONTENTS.

ORIGINAL AND SELECTED ARTICLES.

Typhoid Pneumonia—Its Treatment, etc., by Alban S. Payne, M.D., 41. Viburnum Prunifolium in Threatened Abortion and in Menorrhagia, by D. B. Nisbet, M.D.; Treatment of Chronic Trigeminal Neuralgia, 47. The Cure of Sciatica and Violent Neuralgia by Hypodermic Injections of Ether, by C. G. Comegs, M.D., 49. On the Use of Certain Caustics, by David W. Yandell, M.D., 51. The Care of the Eyes, 54. Opium in Peritonitis, by R. M. Hand, M.D., 55. On the Use of Chloral Hydrate Enemata, 56. Phosphorus, combined with Malaria, the Cause of Yellow Fever, 57.

ABSTRACTS AND GLEANINGS.

Alkaloids of Opium, 58. Oxalate of Cerium in Chronic Cough; Brunella Vulgaris, 59. Tubercular Meningitis, 60. Antiseptic Dressings, 61. An Ulcer Nussbaumed; Syphilis, 62. Antiseptic Gauze; Apiol, 63. How to Avoid Leaving Scars; Injurious Effect of Use of Tobacco on the Eye; Chronic Bright's Disease, 64. Intestinal Gas; Electricity for Sleeplessness; Hooping-cough and Ulceration of the Phrænum, 65. Vinegar Fumes in Bronchitis; Treatment of Tetanus; Treatment of Gingivitis; Salicylic Acid, 66. Treatment of Cold Feet; Evacuation of Pus from the Pleura; Turpentine Vapor Baths; Polypi of the Ear; Subnitrate of Bismuth, 67. Articular Rheumatism; Syphilitic Laryngitis; Charcoal for Burns; Horrible, 68. Cholera Infantum; Carbolic Acid Spray in Diphtheria; Blepharitis; Insomnia, 69. Fibroid Tumors of the Uterus; Codeia; Diphtheria; Ulcers; Quinine; Gonorrhœa; Veratrum, 70.

SCIENTIFIC ITEMS.

New Inventions; The Perfection of Nature; Kidney Worm; A Costly Telescope, 71. An Exhibition of Recent Inventions; Whirlwinds; Delicate Test; Blasting Agent, 72.

PRACTICAL NOTES AND FORMULÆ.

After-Pains; Herpes Circinatus; Bleeding Hemorrhoids; Bromo-Hydrate of Quinine, 73. Hair Dyes; Burns; Dyspepsia; Pulmonary Alterative Pills; Spirits of Nitre Poisonous, 74. Gonorrhœa; Baldness; Hawk-weed; Cough Mixture; Lactopeptine; Syrup of Chloral, 75. Frosted Feet and Hands; Paresi's Hæmostatic Collodion; Tinctura Saponis Viridis; Spermatorrhœa; Cancer; Shock; Peach Leaves, 76.

EDITORIAL AND MISCELLANEOUS.

Medico-Chirurgical Society; Southern Medical College; New California Remedies; The Plague in Russia, 77. The Vicksburg Physicians; Department of Public Health, 78. S. C. Medical Society; Locals, 79. Book Notices; Special Notices, 80.

